# Effect of chitosan on buck semen quality and semen plasma metabolites during low-temperature storage

**DOI:** 10.3389/fvets.2025.1544234

**Published:** 2025-03-13

**Authors:** Meijun Song, Bingke Jia, Dinghui Dai, Xiaoli Xu, Jiaxue Cao, Jiazhong Guo, Linjie Wang, Tao Zhong, Siyuan Zhan, Li Li, Hongping Zhang

**Affiliations:** ^1^Key Laboratory of Livestock and Poultry Multi-Omics, Ministry of Agriculture and Rural Affairs, College of Animal Science and Technology, Sichuan Agricultural University, Chengdu, China; ^2^Farm Animal Genetic Resources Exploration and Innovation Key Laboratory of Sichuan Province, Sichuan Agricultural University, Chengdu, China

**Keywords:** chitosan, buck semen, seminal plasma metabolites, low-temperature preservation, fatty acyls

## Abstract

**Background:**

Optimizing buck semen preservation techniques can significantly advance the goat industry. This study aimed to investigate the effects of chitosan on sperm quality and seminal plasma metabolite profiles in bucks during low-temperature storage at 4°C.

**Results:**

The results showed that when 0.2 mg/mL chitosan was added to semen dilution, sperm viability and antioxidant capacity were highest and significantly higher than the control group (*p* < 0.05). Sperm viability decreased progressively with increasing storage time at 4°C. However, on day 5, sperm viability was significantly higher in all groups where chitosan was added to the semen dilutions than in the control group (*p* < 0.05). A total of 23 classes of metabolites were detected in the non-targeted metabolism group of seminal plasma. The metabolite caused by chitosan mainly included fatty acyls, phospholipids, amino acids and organic acids. Most differential metabolites in fatty acyls and glycerophospholipids in chitosan-treated semen were decreased and enriched in the anabolic pathway of unsaturated fatty acids. Additionally, several oligopeptides showed correlations with sperm quality.

**Conclusion:**

These results suggest that adding 0.2 mg/mL chitosan to semen diluent successfully prolongs the low-temperature preservation of semen mainly by altering the anabolism of lipids and amino acids. This provides theoretical support and practical reference for the applying chitosan in the low-temperature preservation of buck semen.

## 1 Introduction

Advancing low-temperature and cryopreservation technologies for male ruminants is crucial for the sustainable development of the livestock industry. Animal sperm preservation includes room temperature, low temperature, and freezing. Room temperature preservation facilitates normal metabolic activities but leads to a rapid decline in sperm vitality due to nutrient depletion and the accumulation of reactive oxygen species (1). Although cryopreservation enables long-term storage, the freezing process significantly damages sperm, reducing the viability of post-thaw samples (2). In contrast, low temperature preserves sperm to several days or sometimes weeks and ensures a high fertilization rate (3, 4). In addition, low temperature allows small-scale germplasm exchange within the adequate preservation time, offering broader application prospects. The regulation of oxidative homeostasis in spermatozoa is critical for sperm preservation. Primarily, it extends low-temperature preservation duration and maintains fertilization competence (5). Chitosan is a biopolymer derived from chitin, a naturally occurring polysaccharide found in the exoskeletons of arthropods such as crustaceans (6). It consists of glucosamine and N-acetylglucosamine units, and is known for its antimicrobial, antioxidant, and biocompatible properties (7, 8). Chitosan reacts with and effectively scavenges free radicals, with its antioxidant activity being closely related to its molecular weight (9). Moreover, the concentration of chitosan and its derivatives is critical for resistance against fungi and bacteria (10, 11). Recent studies have shown that higher quality post-thaw buck semen can be achieved by supplementing the semen diluent with 15 μg/mL of chitosan nanoparticles prior to cryopreservation (12). Therefore, chitosan’s antioxidant and antimicrobial properties are expected to enhance the effectiveness of semen preservation and artificial insemination techniques in bucks.

Metabolomic analysis investigates dynamic changes in the composition, structure, and concentration of metabolites in spermatozoa, providing critical insights into the metabolic factors associated with sperm freezing and thawing, and enabling the prediction of semen quality and post-thaw survival (13, 14). Compared to transcriptomics and proteomics, metabolomics is less affected by post-transcriptional modifications and provides insights into spermatozoa’s metabolic characteristics, biological functions, and environmental adaptations (15). Non-targeted metabolic analysis of bull spermatozoa revealed a richness in organic and fatty acids, with metabolites such as *γ*-aminobutyric acid, carbamate, benzoic acid, lactic acid, and palmitic acid identified as potential biomarkers of fertility (16).

The antioxidant capacity of spermatozoa is crucial for the successful preservation of animal semen. Here, different concentrations of chitosan were added to buck semen diluents to detect changes in sperm quality and antioxidant properties on low-temperature preservation. Metabolomic assays using Liquid Chromatography-Mass Spectrometry (LC–MS) non-targeted metabolomics were performed to screen for different metabolites among groups. This study investigated the effects of chitosan on semen viability and antioxidant capacity in bucks, uncovering the underlying mechanism by which chitosan enhances sperm preservation through the modulation of fatty acids, phospholipids, amino acids, and organic acid metabolites.

## 2 Materials and methods

### 2.1 Buck rearing conditions and semen collection

The semen used in this experiment was obtained from Tianfu Goat at the Sichuan Agricultural University breeding farm (Ya’an, Sichuan, China). The animal study was approved by Sichuan Agricultural University (No. DKY-SR230616). Five bucks aged 1–2 years with similar body conditions were selected. The testes of the bucks were smooth, without lumps, nodules, or localized hardening, and there were no signs of cryptorchidism or size disparity. All bucks exhibited normal libido. Each buck was housed individually at room temperature under identical conditions, fed oat grass, broad bean hulls, and concentrates every morning and evening, along with regular immunization and deworming. Semen collection was performed four times a week between 7:00 and 8:00 am using the artificial vagina method. The experiment was conducted in the autumn and lasted for 4 weeks. After semen collection, the ejaculate volume ranged from 0.5 to 1.5 mL, with a density not lower than 1.5 × 10^^9^ sperm/mL. It was grayish-white, odorless, and was stored in a 37°C water bath and diluted within 30 min.

### 2.2 Preparing semen dilution

TRIS (Wuxi Tongchuang Biotechnology CO., Ltd., BCR0001, Jiangsu, China) 3,630 mg, glucose 1,000 mg, fructose (Sangon, A600213-0500, Shanghai, China) 1,140 mg, sodium bicarbonate (Solarbio, S5240, Beijing, China) 125 mg, citric acid (Solarbio, C8610, Beijing, China) 1820 mg, HEPES (Solarbio, H8090, Beijing, China) 600 mg, potassium penicillin 100,000 IU (Healton, Chengdu, China), streptomycin sulfate 100,000 IU (Healton, Chengdu, China), and 5 mL of egg yolk was dissolved in 100 mL of distilled water. The mixture was stirred with a magnetic stirrer for 10 min, then sealed with a film and stored at 4°C. For chitosan preparation, 0.1 g of chitosan powder ((C6H11NO4)n) (Solarbio, C8320, Beijing, China) (deacetylation degree >90%) was dissolved in 10 mL of the base dilution solution to prepare a 10 mg/mL preservation solution, which was also sealed and stored at 4°C.

### 2.3 Diluting buck semen with various chitosan concentrations

Semen and diluent were preheated in a 37°C water bath, diluted isothermally 10-fold using the gradual dilution method to avoid the “dilution effect” and protect sperm viability. Firstly, 100 μL of semen and diluent were transferred to a 1.5 mL centrifuge tube for 1:1 initial dilution, stood for 5 min. Then, 400 μL of diluent was added for 1:5 secondary dilution. And finally, another 400 μL of diluent was added to complete the 10-fold dilution. The diluted semen was divided into 6 groups. Chitosan dilutions of 0, 5, 10, 20, 40 and 80 μL were added to each 1 mL of base dilution, resulting in final chitosan concentrations of 0, 0.05, 0.1, 0.2, 0.4 and 0.8 mg/mL. Three replicates were designed for each group. The diluted semen was stored at a constant temperature of 4°C. It was gently inverted and mixed twice daily, at 8:00 am and 8:00 pm, to prevent sperm from settling at the bottom of the centrifuge tubes.

### 2.4 Sperm motility analysis after low-temperature

Before the test, the stage and normal slides of the computer assisted semen analysis (CASA) system (Minitube, AndroVision, Tiefenbach, Germany) were preheated to 37°C. A 50 μL semen sample was pre-warmed in a 37°C water bath for 10 min. A 3 μL aliquot of semen was then placed onto a pre-warmed standard glass slide (25.4 mm × 76.2 mm) and covered with a cover slip (20 mm × 20 mm) (Citotest, Nantong, China). The use of a classic slide system with pre-warmed slides and cover slips minimized potential errors related to sample evaporation and motility measurement variability, as recommended for standardized CASA protocols (17). Sperm viability and motility were assessed every 24 h for 5 days. Sperm motility parameters, including straight-line velocity (VSL, μm/s), curvilinear velocity (VCL, μm/s), average path velocity (VAP, μm/s), beat cross frequency (BCF, Hz), Straightness (STR, VSL/VAP, %) linearity (LIN, VSL/VCL, μm/s), and wobble (WOB, VAP/VCL, %), were measured using CASA by aspirating 3 μL of semen from each low-temperature groups on days 1, 3, and 5. Three fields with over 200 spermatozoa were randomly selected from each group for observation. The test was repeated three times, and the average value was used as the final result.

### 2.5 Sperm plasma membrane integrity

Sperm membrane integrity was evaluated using the hypo-osmotic test method (18). Sodium citrate (0.735 g) and fructose (1.351 g) were dissolved in 100 mL of purified water to create a hypotonic solution stored at 4°C. After 5 days of low-temperature storage, 100 μL of semen was aspirated and added to 900 μL of hypotonic solution, then incubated in a water bath at 37°C for 30 min. Plasma membrane integrity was assessed by observing sperm swelling under low osmotic pressure with a microscope (Murzider, MSD1125, Shenzhen, China). Three fields with over 200 spermatozoa were randomly selected from each group for observation. The test was repeated three times, and the average value was used as the final result. The proportion of tail-bent spermatozoa to the total spermatozoa was calculated as the plasma membrane integrity rate.

### 2.6 Sperm antioxidant property measurement

The assays were performed on days 1, 3, and 5 according to the instructions of the T-AOC assay kit (Solarbio, BC1315, Beijing, China), CAT assay kit (Solarbio, BC0205, Beijing, China), ROS assay kit (Solarbio, CA1410, Beijing, China), and MDA assay kit (Solarbio, BC0025, Beijing, China). For T-AOC measurement, 1 mL of pre-cooled extraction solution was added per 5 million spermatozoa, followed by ultrasonic disruption. The sample was then centrifuged at 10,000 rpm for 10 min at 4°C, and the supernatant was collected. The absorbance was measured at a wavelength of 593 nm using a microplate reader, and T-AOC was calculated according to the instructions provided in the kit. For CAT and MDA, 1 mL of extract was added to each 5 million spermatozoa, broken by ultrasonication, and then centrifuged at 8000 g for 10 min at 4°C. The supernatant was taken and the absorbance was measured using an enzyme marker at 240 nm, 532 nm and 600 nm, respectively. CAT and MDA were calculated according to the formula in the instruction manual. For ROS, the sperm concentration was adjusted at 5 million spermatozoa/mL, and DCFH-DA was added to make the final concentration at 1–10 μmol/L. The plate was incubated for 20 min at 37°C away from light, and 200 μL of resuspension was aspirated and added to a 96-well plate, and detected by using a fluorescence enzyme marker, setting the excitation wavelength at 488 nm and the emission wavelength at 525 nm, and detecting the intensity of fluorescence before and after stimulation. For each experiment, three biological replicates were performed, with three technical replicates each.

### 2.7 Sperm non-targeted metabolomic analysis

The semen was divided into control groups (CT, *n* = 6) and 0.2 mg/mL chitosan-treated groups (CS, *n* = 6) and subjected to non-targeted metabolomic analysis on days 1 and 5 of low temperature. Low-temperature semen was centrifuged at 4000 r/min, 4°C for 5 min. A 40 mg sample was added to a steel ball, homogenized using a ball mill for 20 s, and centrifuged at 3000 r/min, 4°C for 30 s to allow the sample to settle to the bottom of the tube. The samples were extracted with methanol and separated using a UPLC (Shimadzu, LC-30A, Kyoto, Japan) and a Waters ACQUITY Premier HSS T3 column. Data acquisition was performed using the information-dependent acquisition (IDA) mode with Analyst TF 1.7.1 Software (Sciex, Concord, ON, Canada). TOF MS scan parameters were: mass range, 50–1,000 Da; accumulation time, 200 ms; dynamic background subtraction, on. Product ion scan parameters were: mass range, 25–1,000 Da; accumulation time, 40 ms; collision energy, 30 or − 30 V in positive or negative modes; collision energy spread, 15; resolution, UNIT; charge state, 1 to 1; intensity, 100 cps; exclude isotopes within 4 Da; mass tolerance, 50 ppm; the maximum number of candidate ions to monitor per cycle, 18.

The original LC–MS data file was converted into mzXML format by ProteoWizard software. Peak extraction, alignment and retention time correction were performed by the XCMS program. The “SVR” method was used to correct the peak area. Peaks with a detection rate lower than 50% in each group were discarded. Metabolic identification information was obtained by searching the laboratory’s self-built database, integrated public database, AI database and metDNA. At the same time, ensure that the CV value of the QC samples is less than 0.3 to ensure the stability and reliability of the analytical results, and then carry out the positive and negative modes for metabolite identification. Finally, extract the substances with a combined score of 0.5 or more and a CV value of less than 0.3 for QC samples, and then merge the positive and negative modes (retaining the substances with the highest qualitative grade and the smallest CV value) to obtain the ALL sample data file.

### 2.8 Statistical analysis

All results were expressed as mean ± standard error of the mean (SEM) and analyzed using SAS Studio software (SAS Studio v3.8, Cary, NC, USA). Experimental data were analyzed using mixed model for repeated measures or one-way ANOVA followed by Tukey–Kramer multiple comparisons. Correlation analysis was performed using the Spearman correlation coefficient. A *p*-value less than 0.05 was considered statistically significant.

## 3 Results

### 3.1 Chitosan improves the viability of buck sperm during low-temperature preservation

Sperm viability was measured every 24 h starting from day 0 (Table 1). At day 0, sperm viability across all chitosan concentrations were insignificantly different (*p* > 0.05). By day 1, viability declined across all treatments; however, sperm viability in the 0.2 mg/mL (*p* = 0.037) and 0.4 mg/mL (*p* = 0.049) chitosan groups remained significantly higher compared to the control group (*p* < 0.05). On days 2–5, sperm viability continued to decrease, yet the 0.2 mg/mL chitosan group maintained higher viability than the control group (*p* < 0.05). In contrast, there was no significant difference in the 0.2 mg/mL chitosan group for 2–4 days of preservation (*p* > 0.05), and a significant decrease in sperm viability was observed on day 5 (*p* < 0.01).

**Table 1:** Viability of buck sperm stored at 4°C under different chitosan concentrations.

Time (day)	0	0.05	0.1	0.2	0.4	0.8 (mg/mL)
0	85.82 ± 0.73^a^	85.80 ± 0.08^a^	85.55 ± 0.39^a^	85.86 ± 0.23^a^	85.46 ± 1.00^a^	86.36 ± 0.88^a^
1	76.51 ± 1.15^cdef^	75.84 ± 1.89^bcde^	77.20 ± 1.18^bcd^	79.80 ± 1.40^b^	79.71 ± 0.48^b^	79.24 ± 1.25^bc^
2	69.44 ± 1.67^ijkl^	71.50 ± 1.92^fghij^	72.22 ± 0.23^efghi^	74.21 ± 1.29^defg^	72.38 ± 1.20^efgh^	72.54 ± 0.77^efgh^
3	67.98 ± 1.38^jkl^	70.14 ± 1.81^ghij^	71.70 ± 0.29^fghij^	74.11 ± 1.04^defg^	71.46 ± 1.34^ghij^	70.82 ± 1.23^ghij^
4	54.43 ± 1.12^p^	64.97 ± 0.52^lm^	67.72 ± 0.92^ijkl^	72.19 ± 0.37^efghi^	69.79 ± 0.18^hij^	61.45 ± 1.34^mn^
5	50.78 ± 0.73^q^	59.94 ± 0.46^no^	63.06 ± 0.47^mn^	69.24 ± 0.32^hijk^	65.16 ± 1.27^klm^	56.69 ± 1.61^op^

Different lowercase letters indicate significant differences (*p* < 0.05) and the same letters indicate no significant differences (*p* > 0.05), the same below.

### 3.2 Chitosan increases sperm motility and plasma membrane integrity during low-temperature storage

**Figure 1:**
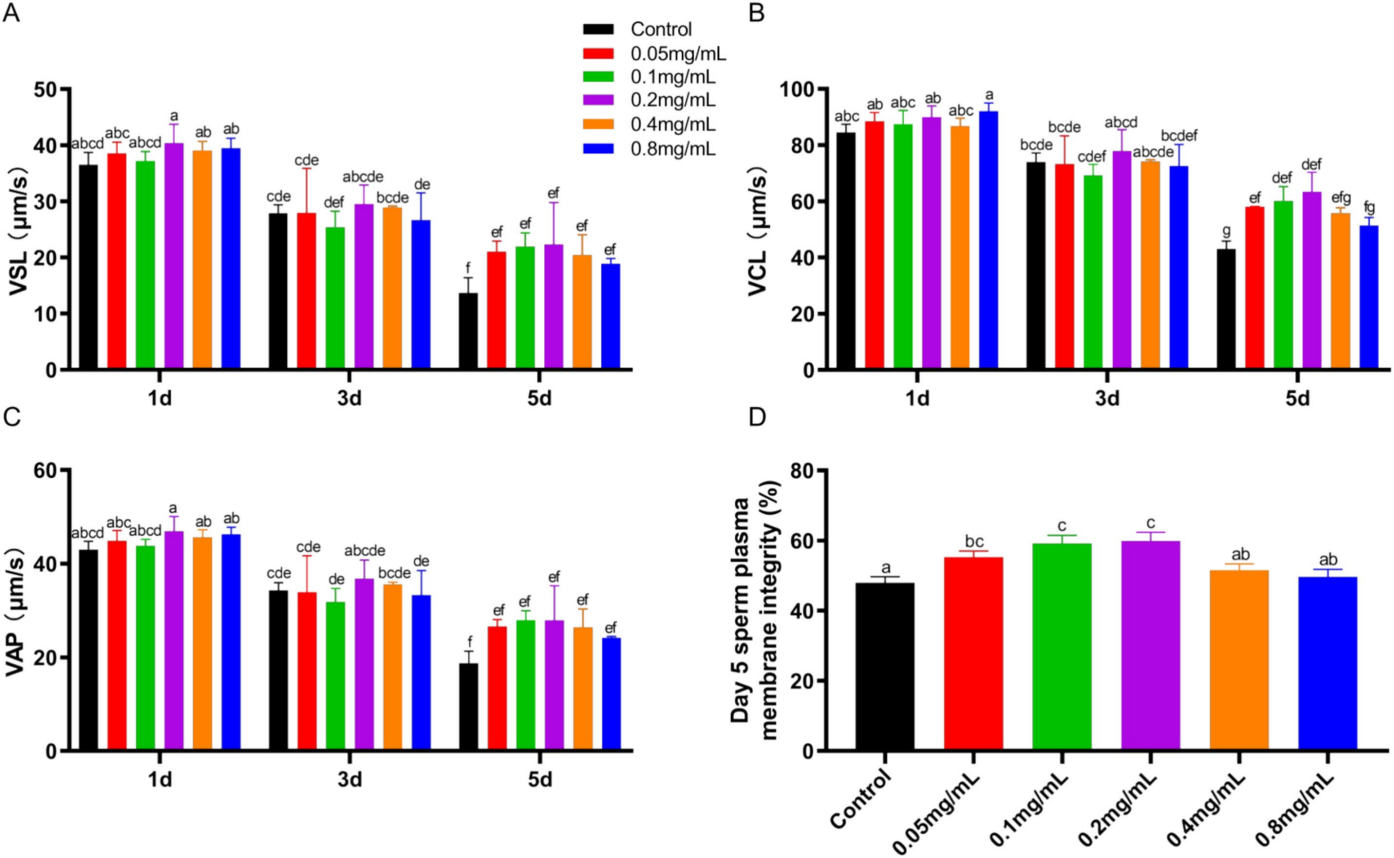
Sperm motility and plasma membrane integrity of bucks affected by different concentrations of chitosan. **(A)** VSL. **(B)** VCL. **(C)** VAP. **(D)** Plasma membrane integrity. VSL, straight-line velocity; VCL, curvilinear velocity; VAP, average path velocity. Different lowercase letters indicate significant differences (*p* < 0.05) and the same letters indicate no significant differences (*p* > 0.05), the same below.

The VSL, VCL and VAP of sperm motility were assessed after adding different concentrations of chitosan to the low-temperature preservation diluent of buck semen (Figures 1A–C). With increasing storage time, all motility parameters (VSL, VCL, VAP) showed a declining trend, which was most pronounced in the control group (*p* < 0.05). VSL and VAP were minimally affected by different concentrations of chitosan, showing no significant difference compared to the control group (*p* > 0.05). The addition of 0.05–0.2 mg/mL chitosan to semen dilutions significantly increased VCL at 5 days compared to the control group (*p* < 0.05). Furthermore, our results indicate that chitosan is most effective at improving motility parameters at low concentrations, while high concentrations (0.8 mg/mL) exhibit an inhibitory effect. While the BCF and LIN remained unaffected by chitosan treatment (*p* > 0.05), significant improvements were observed in the WOB and STR (*p* < 0.05) (Supplementary Figures S1A–D). On day 5 of low-temperature storage, sperm plasma membrane integrity was significantly higher in the 0.05 (*p* = 0.005), 0.1 (*p* = 0.001), and 0.2 (*p* < 0.001) mg/mL chitosan-treated groups compared to the control (*p* < 0.05) (Figure 1D). These results indicate that chitosan, particularly at low concentrations, effectively preserves buck sperm motility during cold storage.

### 3.3 Improvement of antioxidant properties of buck semen by chitosan during low-temperature storage

**Figure 2:**
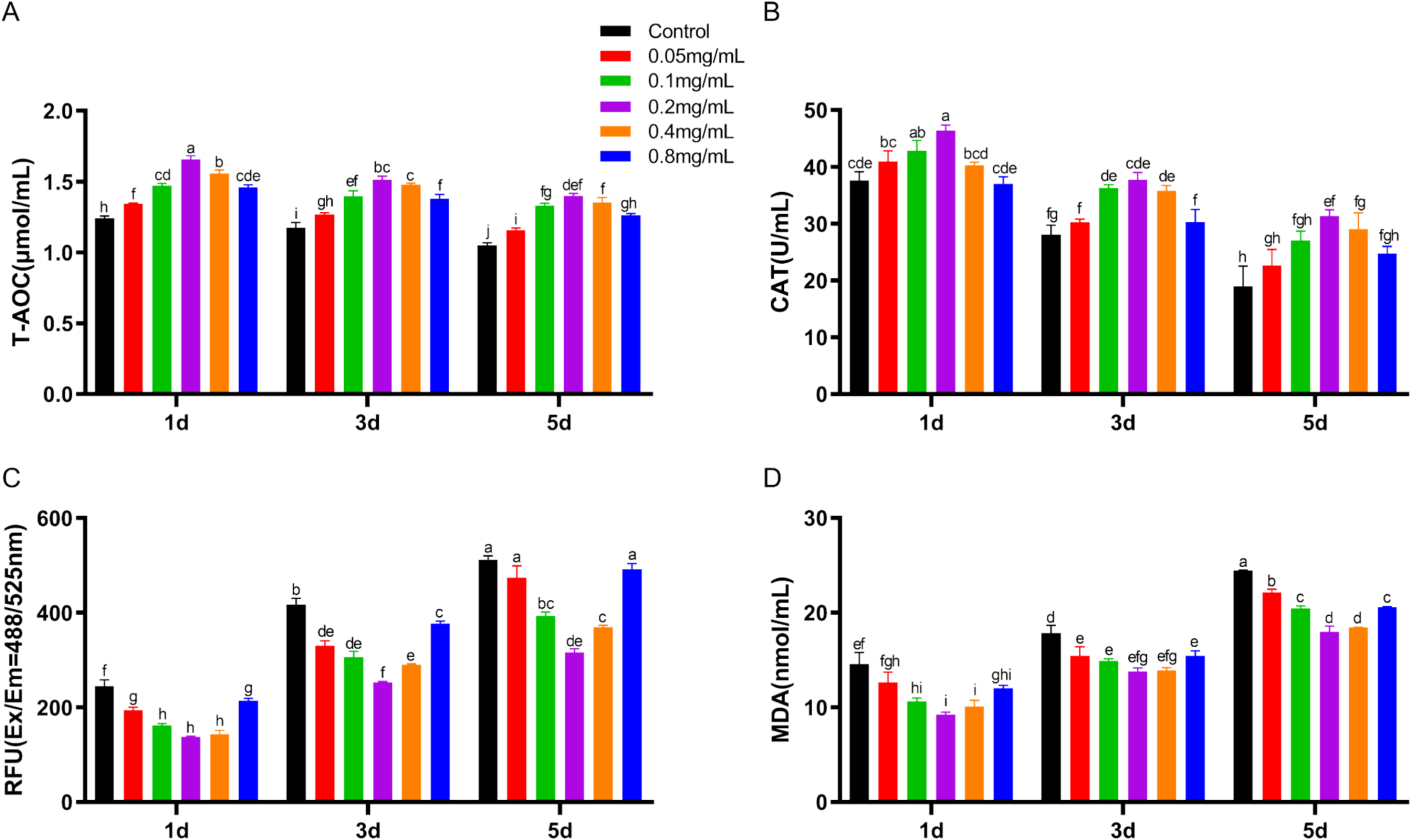
Effect of chitosan on the antioxidant properties of bucks semen stored at low temperature. **(A)** The levels of T-AOC. **(B)** The levels of CAT. **(C)** The levels of ROS. **(D)** The levels of MAD.

During low-temperature preservation, T-AOC and CAT levels in spermatozoa showed a gradual decrease. These levels initially increased and then decreased with increasing chitosan concentration (Figures 2A,B). On day 1, the 0.2 mg/mL chitosan group showed a significantly higher T-AOC compared to the control, with this enhancement maintained through days 3 and 5 (*p* < 0.05). Similarly, the 0.2 mg/mL chitosan group had higher CAT activity on day 1 compared to the control (*p* < 0.05), a trend that persisted on days 3 and 5. ROS and MDA levels increased with preservation time (Figures 2C,D). Low concentrations of chitosan reduced ROS and MAD levels, with 0.2 mg/mL showing the most pronounced effect, while 0.8 mg/mL had significantly reduced ROS inhibition compared to 0.2 mg/mL. The findings demonstrate that the addition of 0.2 mg/mL chitosan to semen dilutions significantly enhances antioxidant capacity and reduces oxidative stress in buck sperm during cold storage. Therefore, the 0.2 mg/mL chitosan group was selected for further analysis.

### 3.4 Differential metabolites in seminal plasma affected by chitosan

**Figure 3:**
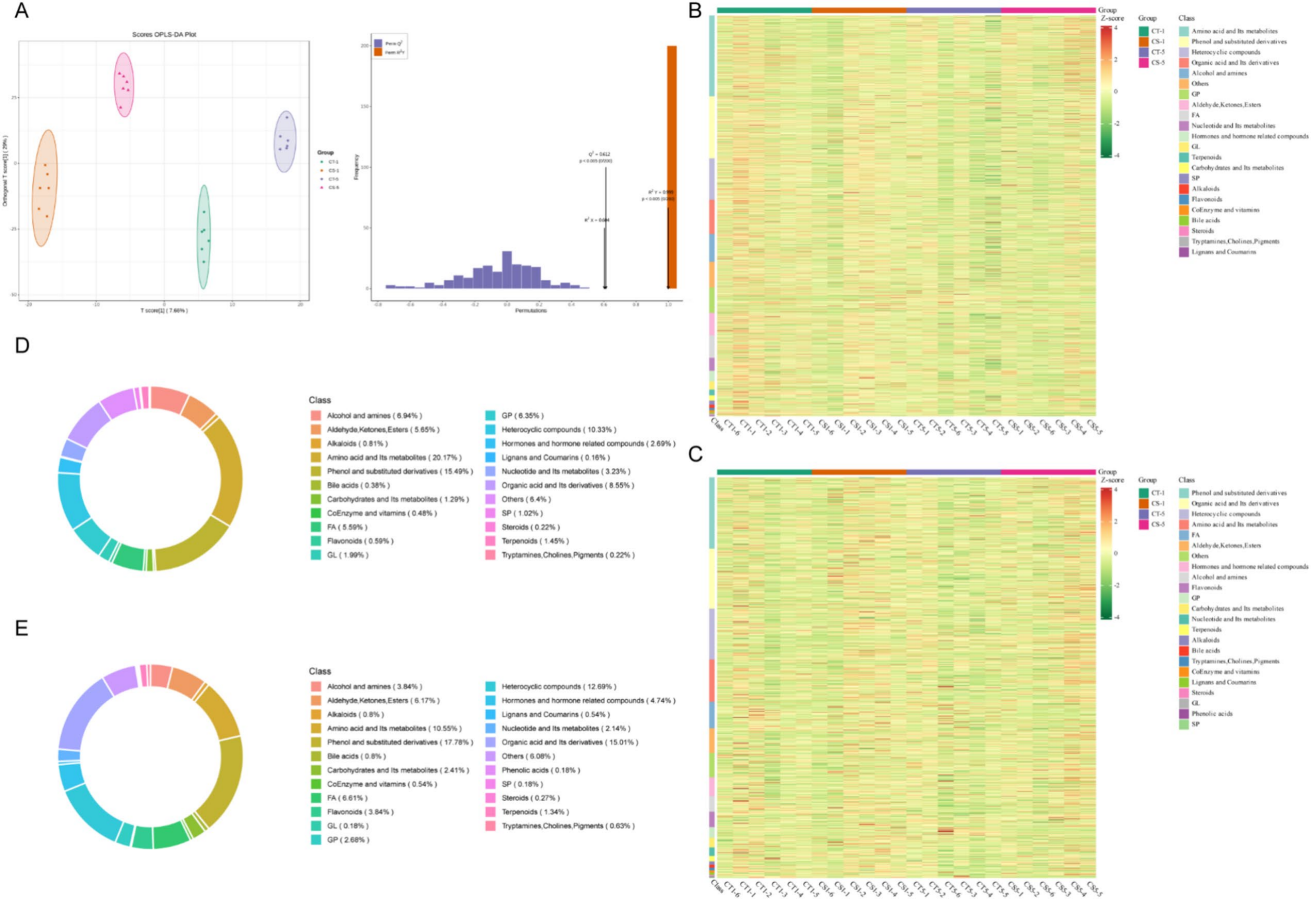
Non-targeted metabolic profiles of seminal plasma from low-temperature preserved buck semen. **(A)** OPLS-DA score plot. **(B)** Heat map of the corresponding metabolic signatures detected in positive ion mode. **(C)** Heat map of the corresponding metabolic signatures detected in negative ion mode. **(D)** Proportion of positive ion mode metabolites. **(E)** Proportion of negative ion mode metabolites. FA, fatty acyl; GL, glycerides; GP, glycerophospholipids; SP, sphingolipid.

Untargeted metabolomic analysis was performed on control and 0.2 mg/mL chitosan-treated groups on days 1 and 5 of low-temperature storage. The results showed a high overlap in total ion flow peak profiles, with consistent retention times, and response intensities. PCA confirmed the stability and reproducibility of the analysis system, with quality control samples clustering tightly (Supplementary Figure S2). Orthogonal partial least squares discriminant analysis (OPLS-DA) further delineated the relationship between metabolites and samples, showing clear separation between treatment groups. This separation underscores the significant metabolite changes induced by chitosan treatment and extended storage (Figure 3A). In both ionization modes, the OPLS-DA models demonstrated robust performance, with high goodness of fit (*R*^2^ = 0.99) and predictive power (*Q*^2^ = 0.71).

**Figure 4:**
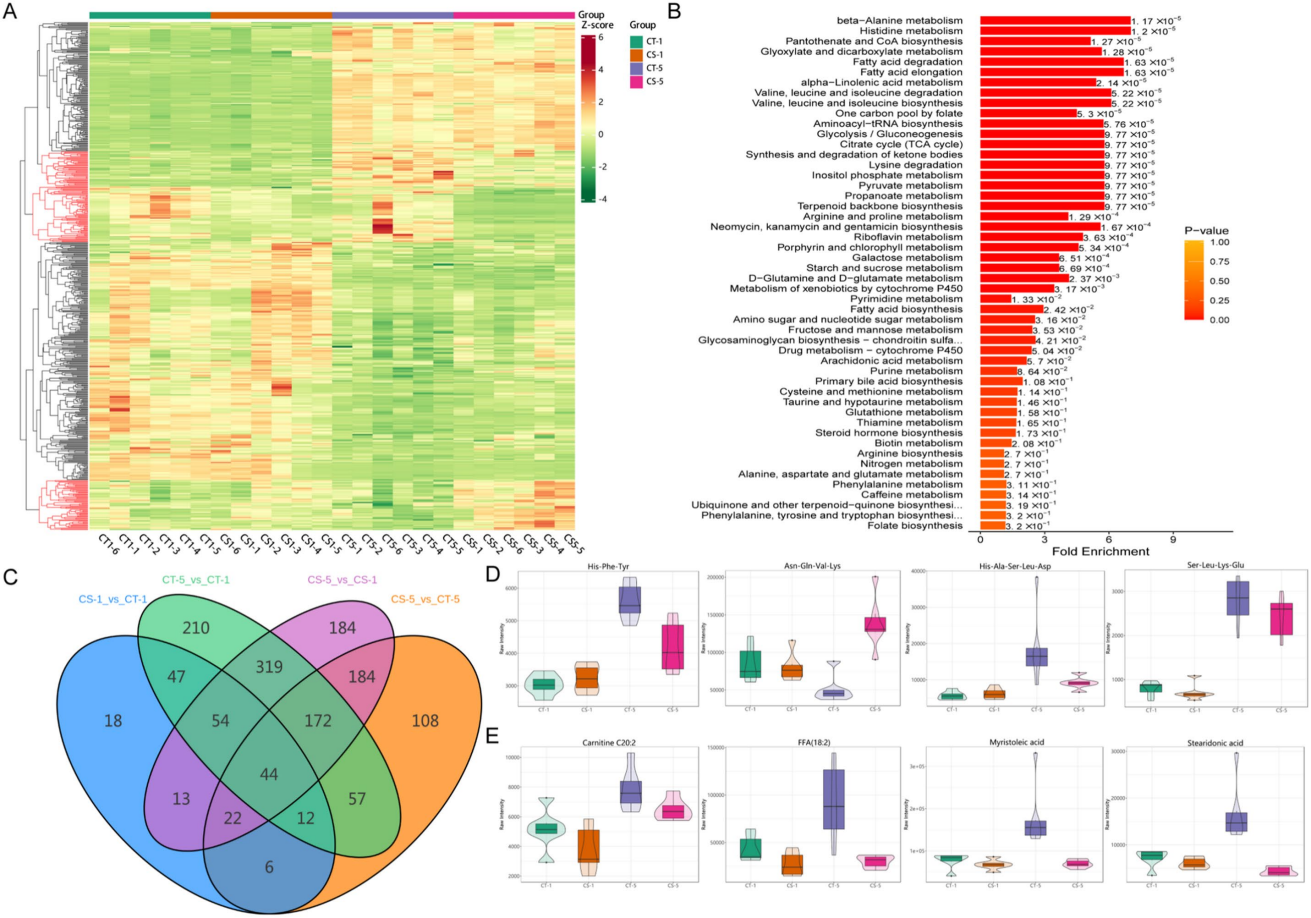
Overall differential metabolite among groups in seminal plasma of bucks. **(A)**: Heat map of overall differential metabolites, differential metabolites at the same number of storage days are shown in red **(A)** marked in red. **(B)** KEGG pathway enrichment analysis of differential metabolites. **(C)** Number of differential metabolites between groups Wayne plots. **(D)** Expression patterns of random oligopeptides. **(E)** Expression patterns of random fatty acyls.

A total of 2,978 metabolites were detected in 20 samples from 4 groups: 1119 in anionic mode and 1859 in cationic mode. In cationic mode, 22 classes of metabolites were detected, dominated by amino acids and their metabolites, phenol and its substituted derivatives, heterocyclic compounds, organic acids and their derivatives, and glycerophospholipids. In anionic mode, 23 classes were detected, dominated by phenol and its substituted derivatives, organic acids and their derivatives, heterocyclic compounds, amino acids and their metabolites, and fatty acyls (Figures 3B–E). The analysis identified over 600 differential metabolites between groups. The most numerous were amino acids and their metabolites, followed by phenol and its substituted derivatives, heterocycles, organic acids, and their derivatives, fatty acyls, and glycerophospholipids (Figure 4A, Supplementary Figures S3A,B, Supplementary Table S1). Most changes were due to low temperature, while a few were caused by adding chitosan, mainly including amino acids and their metabolites, fatty acyls and glycerophospholipids (Figure 4A).

Kyoto Encyclopedia of Genes and Genomes (KEGG) enrichment analysis revealed significant enrichment of differential metabolites in amino acid and fatty acid metabolic pathways (Figure 4B, Supplementary Figure S3B). The lowest number of differential metabolites was observed in the control and treatment groups on the first day of low-temperature storage. Five days of storage had a greater effect on the number of differential metabolites than the addition of chitosan. Differential metabolite analysis between the groups revealed 44 common differential metabolites, of which 21 were amino acids and their metabolites, fatty acyls, and glycerophospholipids (Figure 4C, Supplementary Table S2). This study found various oligopeptides in semen plasma metabolites, which were differentially expressed with the addition of chitosan or during low-temperature preservation (Figure 4D). Most differentially expressed fatty acyl metabolites increased in CT5 compared to other groups (Figure 4E). This study confirmed that metabolite levels in seminal plasma changed with increased low-temperature preservation time. We focused on the effect of chitosan on semen low-temperature preservation and found more significant between the control and treated groups on day 5. Both differential metabolite and KEGG enrichment indicated that fatty acids and amino acids were key differential metabolites.

### 3.5 Chitosan affects the metabolism of fatty acyl and glycerophospholipids in seminal plasma

**Figure 5:**
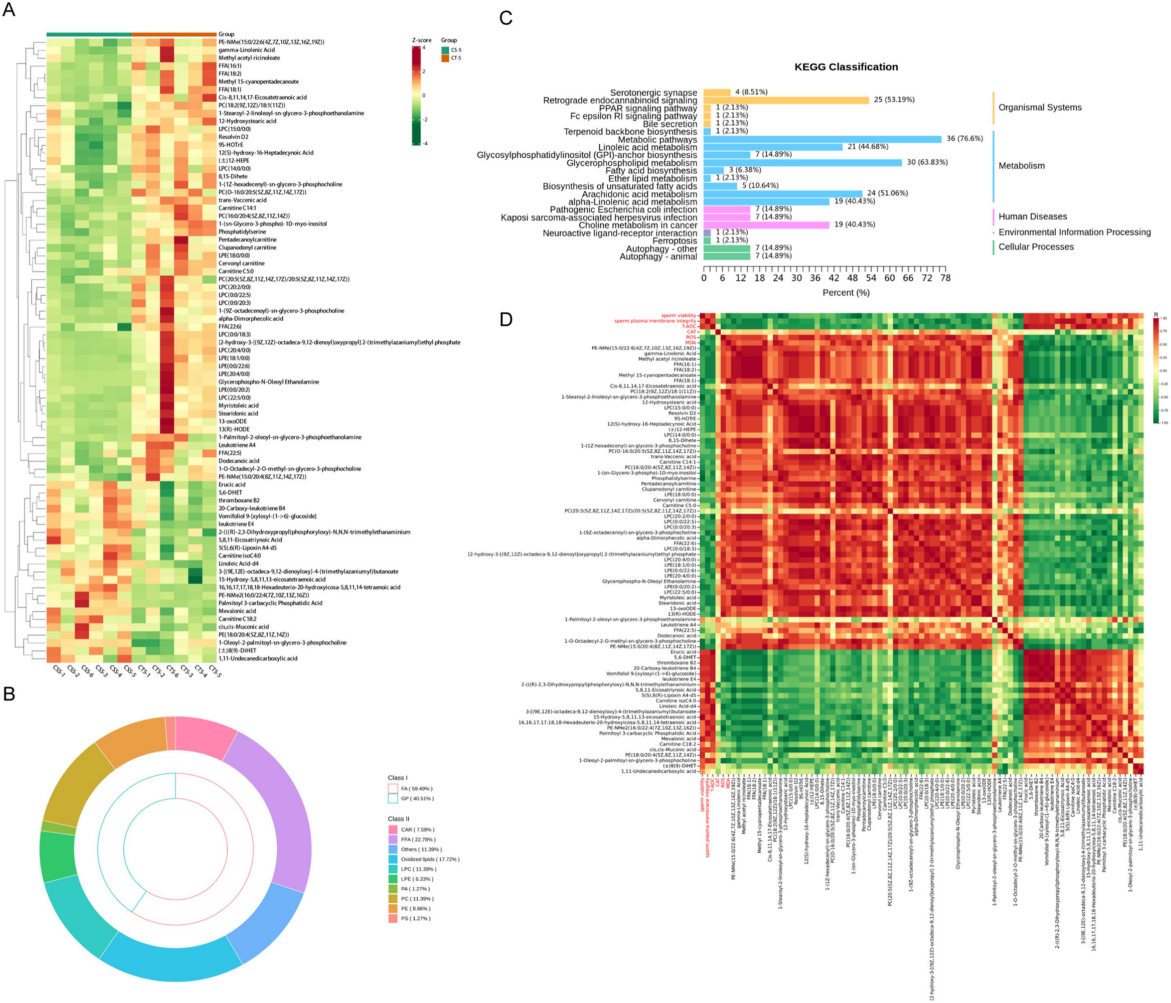
Differential analysis of fatty acyl and glycerophospholipid metabolites in buck semen between CS-5 and CT-5. **(A)** Differential metabolites clustering heat map. **(B)** FA and GP metabolites subclass composition. **(C)** KEGG pathway enrichment analysis of differential metabolites. **(D)** Analysis of the correlation between differential metabolites and sperm quality.

On the fifth day after the addition of chitosan, substantial changes in metabolites of fatty acyls and glycerophospholipids were observed. Therefore, data from day 5 of storage were selected for subsequent analysis. Examination of glycerophospholipids and fatty acyls revealed that the majority of differential metabolites were prominently expressed in the CT-5 group. These metabolites were enriched in pathways related to the synthesis and metabolism of unsaturated fatty acids, including linoleic acid and arachidonic acid metabolism, as well as unsaturated fatty acid biosynthesis (Figures 5A,C). Free fatty acids (FFA) and oxidized lipids comprised the largest proportion of glycerophospholipids and fatty acyls (Figure 5B). Correlation analyses showed that metabolites highly expressed in the CT-5 group were negatively correlated with sperm viability, plasma membrane integrity, and T-AOC, but positively correlated with ROS and MDA levels (Figure 5D). Notably, 5,8,11-eicosatriynoic acid was positively correlated with sperm plasma membrane integrity, while erucic acid was positively associated with T-AOC. Additionally, FFA (18:1) correlated positively with ROS and MDA. These metabolites are all unsaturated fatty acids.

### 3.6 Chitosan affects the metabolism of amino acids and organic acids in seminal plasma

**Figure 6:**
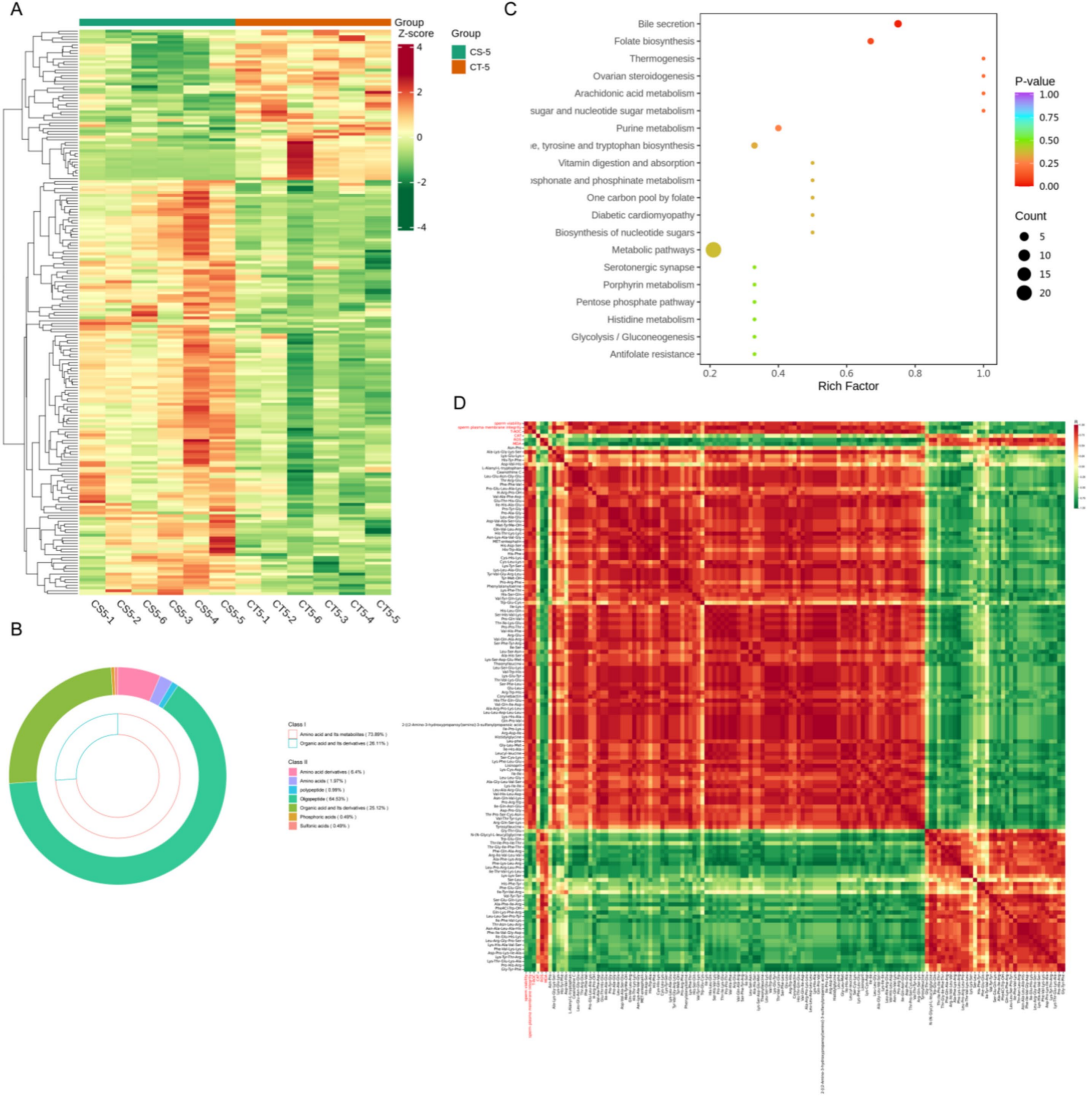
Differential analysis of amino and organic acid metabolites in buck semen between CS-5 and CT-5. **(A)** Differential metabolites clustering heat map. **(B)** Amino acid and organic acid metabolites subclass composition. **(C)** KEGG pathway enrichment analysis of differential metabolites. **(D)** Analysis of the correlation between differential metabolites and sperm quality.

Subsequent analysis of amino acids and organic acids revealed that most differential metabolites were oligopeptides, which showed elevated levels in CS-5 (Figures 6A,B, Supplementary Table S3). This indicates that chitosan is involved in the biosynthesis and metabolism of numerous oligopeptides. KEGG analysis of the differential metabolites showed significant enrichment in bile secretion and folate biosynthesis pathways (*p* < 0.05) and most of the metabolites were enriched in the metabolic pathway. (Figure 6C). Certain oligopeptides exhibit antioxidant and antimicrobial properties (19, 20). These oligopeptides were analyzed for correlation with sperm viability, plasma membrane integrity and ROS-related indices. The highly expressed oligopeptides in CS-5 exhibited positive correlations with sperm viability and plasma membrane integrity and negatively correlated with ROS and MAD (Figure 6D). This suggests that these oligopeptides play a role in reducing oxidative stress, thereby contributing to the maintenance of sperm quality.

## 4 Discussion

Buck spermatozoa are highly susceptible to oxidative stress during storage, severely impacting sperm function. To address this challenge, antioxidants have been widely explored for their ability to prolong sperm viability *in vitro* (21, 22). Chitosan exhibits strong antioxidant properties and excellent biocompatibility (23), making it less prone to rejection and providing a rationale for its inclusion in buck semen diluents. In this study, the addition of chitosan during low-temperature preservation of buck semen was effective in improving sperm viability and mitigating the decline in sperm antioxidant capacity. However, this effect did not scale with increasing concentrations of chitosan; instead, concentrations exceeding 0.4 mg/mL resulted in diminished effectiveness. These findings align with chitosan addition in porcine semen cryopreservation, demonstrating significantly lower lipid peroxidation levels in the chitosan-treated group than in the control group (24). Furthermore, previous studies have highlighted the potential of chitosan and its derivatives as carriers for antioxidant and anti-inflammatory agents (22, 25), underscoring its versatility in applications in biomedicine (26), agricultural science and technology (27), and food industry (28). In addition, dietary chitosan supplementation at a level of 2.5 g/kg for an 8-week interval prior to semen collection plays a critical role in mitigating the negative effects of oxidative stress in the seminal plasma of bucks fed either a control or high-fat diet (29). These findings further support the role of chitosan in improving both the quality and antioxidant capacity of sperm, which could have valuable implications for enhancing reproductive outcomes in buck breeding.

In this study, spermatozoa were stored at 4°C, successfully mitigating the detrimental effects of cryopreservation while maintaining sperm functionality. Previous studies have shown that adding Y-27632 to semen diluents during low-temperature storage significantly improved sperm viability and integrity over 21 days (30). Although the present study only extended to day 5 of low-temperature preservation-a relatively short duration—it provided preliminary evidence suggesting that chitosan enhances semen preservation under low-temperature conditions. Metabolomics in reproductive biology is deemed a more precise approach for delineating phenotypes than transcriptome or proteome (15). This study identified a total of 23 metabolite classes via seminal plasma metabolomics, predominantly comprising phenol and substituted derivatives, amino acid and its metabolites, and organic acid and its derivatives. Importantly, chitosan supplementation during low-temperature preservation led to significant shifts in seminal plasma metabolite profiles, with 216 differential metabolites identified on day 1, increasing to 605 on day 5. This trend aligns with observations in sheep, where longer storage durations yielded even greater numbers of differential metabolites, surpassing those observed with other compounds like Y-27623 (30). These findings suggest that storage duration plays a critical role in driving metabolic changes in seminal plasma, potentially influencing sperm preservation outcomes.

Fatty acids and phospholipids are essential components of cell membranes, and spermatozoa depend on the integrity of the plasma membrane for survival and progressive motility (31). However, the high susceptibility of unsaturated fatty acids to oxidative damage during storage poses a significant challenge to sperm preservation (32–34). In this study, chitosan supplementation reduced unsaturated fatty acid levels after 5 days of low temperature preservation. KEGG pathway analysis revealing an enrichment of metabolites in the unsaturated fatty acid biosynthesis pathway, suggesting that chitosan may modulate lipid metabolism by influencing key enzymes involved in fatty acid synthesis or degradation. Specifically, the reduction in gamma-linoleic acid, a derivative of linoleic acid, could indicate that chitosan acts to prevent the oxidation of polyunsaturated fatty acids, which are highly prone to lipid peroxidation. Recent studies have shown that supplementation with unsaturated fatty acids, such as linoleic, oleic and palmitoleic acids, may increase sperm viability and mitochondrial activity during fluid storage at lower temperatures (6°C), a process that reduces oxidative stress and lipid degradation (35). These fatty acids may be metabolically incorporated into sperm membranes, and the addition of chitosan modulates fatty acid metabolism to maintain sperm viability by reducing oxidative damage.

In addition to lipid metabolism, chitosan supplementation induced significant alterations in amino acid and organic acid pathways. KEGG analysis revealed enrichment in bile acid and folate anabolic pathways, both essential for maintaining sperm function. Folic acid, in particular, is associated with reduced sperm DNA fragmentation and enhanced membrane stability, emphasizing the importance of metabolite composition in sperm quality (36, 37). Additionally, many oligopeptides were identified among the differential metabolites of this study. Oligopeptides exhibit antioxidant, antibacterial, and anti-inflammatory activities while also being characterized by hypoallergenicity, high bioavailability, targeting, and safety (38, 39). Chitosan is widely used for its excellent antimicrobial and antioxidant properties. In this study, chitosan addition led to higher levels of many oligopeptides. Ala-Gln and Gly-Gln increased sperm viability in an ammonia-containing medium during *in vitro* sperm culture (40). In yeast cells, various dipeptides regulate cell membrane function by affecting membrane integrity and lipid composition. Additionally, dipeptides act as antioxidants to reduce MDA levels (41). Notably, specific oligopeptides, such as Phe-Asp-Gly-Asp-Phe, interact with antioxidant enzymes like superoxide dismutase (SOD), altering its conformation to enhance activity (42). These findings are consistent with the enhanced antioxidant capacity observed in chitosan-treated spermatozoa, pointing to a potential link between oligopeptide regulation and oxidative stress mitigation. However, the sheer diversity of oligopeptides presents challenges in elucidating their precise roles.

This study has several limitations that warrant further exploration. While the addition of 0.2 mg/mL chitosan to the semen diluent resulted in notable changes in lipid and amino acid metabolism, analyzing metabolites at a single time point limits insights into the dynamic metabolic changes occurring during preservation. Future studies should explore its long-term preservation effects, elucidate its mechanisms of action, and investigate possible synergistic interactions with other bioactive compounds to further optimize semen preservation techniques. Moreover, although the findings highlight improvements in sperm motility parameters and antioxidant capacity at low temperatures, these results are confined to *in vitro* conditions. The absence of *in vivo* validation restricts the applicability of these findings to practical semen cryopreservation protocols. Expanding research to include in vivo studies and exploring the synergistic effects of chitosan with other compounds could establish a stronger foundation for its use in reproductive biotechnology. Finally, considering the observed alterations in metabolite profiles, uncovering the precise mechanisms by which chitosan influences lipid and amino acid metabolism will be essential. This could involve investigating its role in maintaining cell membrane stability and mitigating oxidative stress. Addressing these gaps will advance our understanding of chitosan’s potential and pave the way for developing optimized semen preservation techniques and improving assisted reproductive technologies.

In summary, this study found that chitosan addition during low-temperature sperm preservation led to significant changes in seminal plasma metabolites, such as fatty acids and amino acids. Chitosan possibly protects spermatozoa by maintaining cell membrane function and scavenging reactive oxygen species. Further studies are needed to investigate the specific roles of fatty acids and oligopeptides in sperm preservation, particularly through the evaluation of different preservation methods and longer storage durations.

## Data Availability

The original contributions presented in the study are included in the article/Supplementary material, further inquiries can be directed to the corresponding authors.
