# Supplementary figures and images for: Effect of chitosan on buck semen quality and semen plasma metabolites during low-temperature storage
Source: Front Vet Sci. 2025 Mar 13;12:1544234. doi: 10.3389/fvets.2025.1544234 (PMC11949143; doi:10.3389/fvets.2025.1544234)

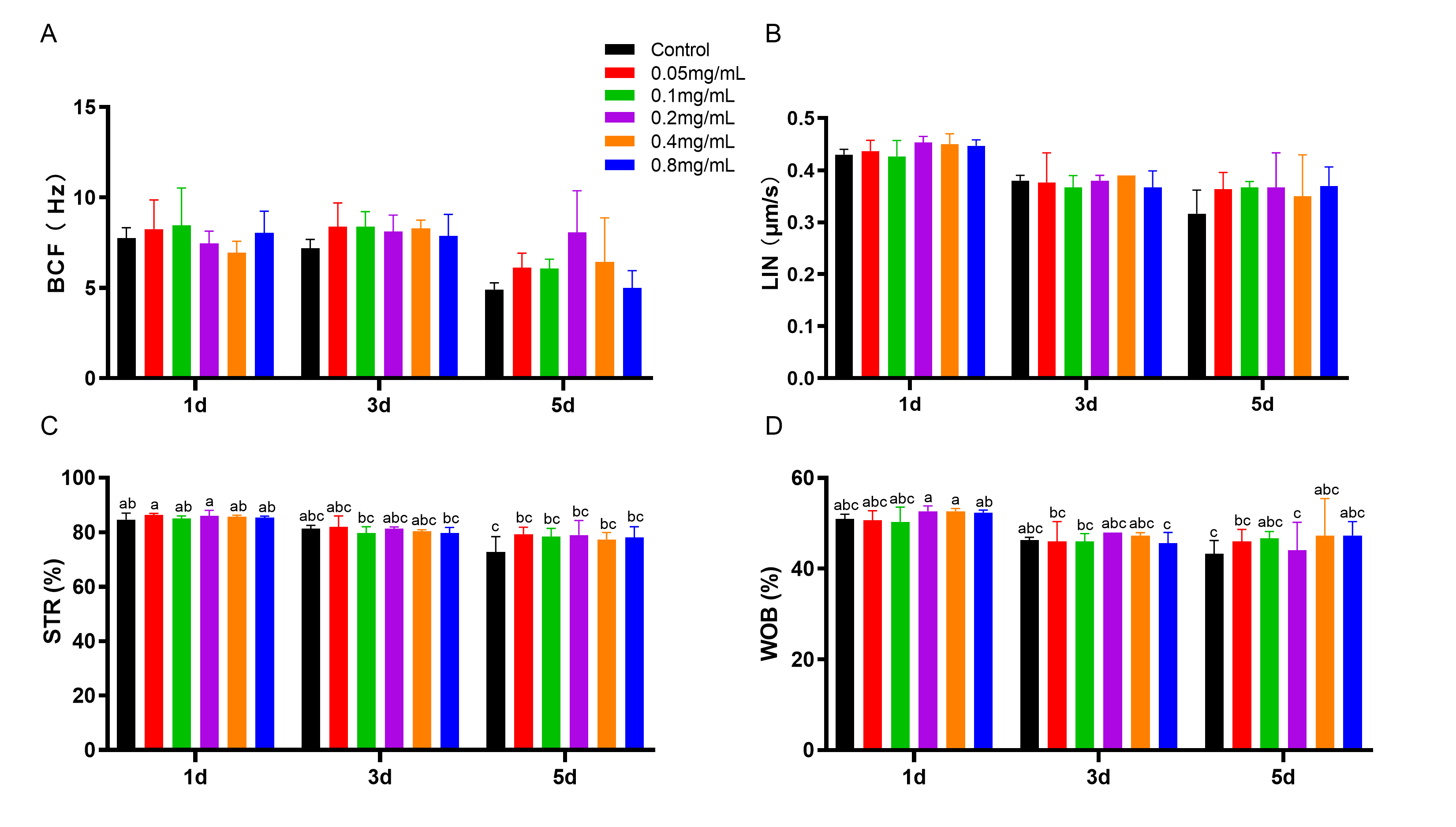

Supplement: Supplementary file 1 [file Image_1.tif]

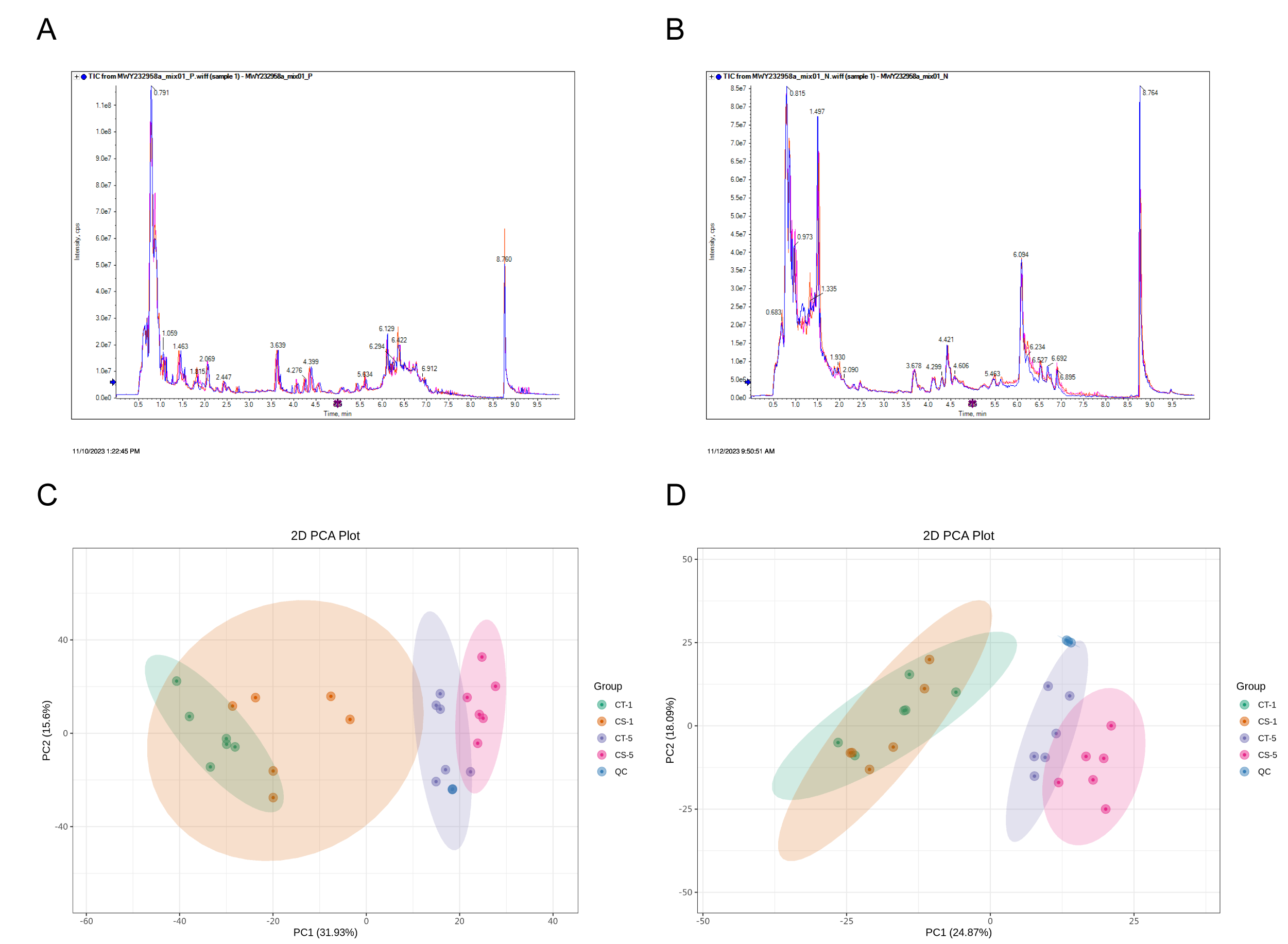

Supplement: Supplementary file 2 [file Image_2.tif]

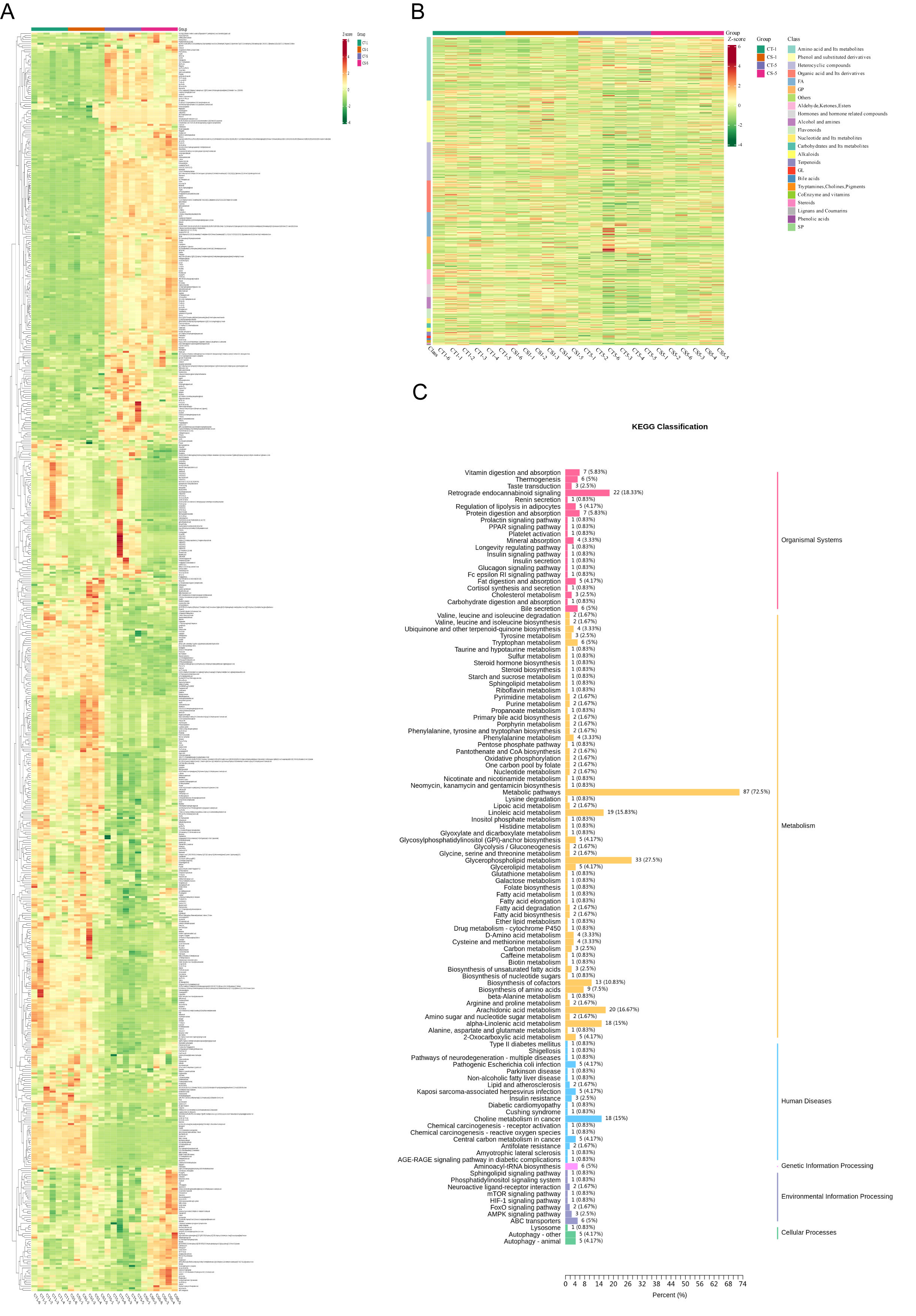

Supplement: Supplementary file 3 [file Image_3.tif]
